# Primary cardiac tumours in infants and children: surgical strategy and long-term outcome

**DOI:** 10.1186/1749-8090-8-S1-O306

**Published:** 2013-09-11

**Authors:** E Delmo Walter, K von Strachwitz, R Hetzer

**Affiliations:** 1 Cardiothoracic Surgery, Deutsches Herzzentrum Berlin, Berlin, Germany

## Background

This is a comprehensive review of our 26-year experience with primary cardiac tumours in children with emphasis on surgical indications, strategies and long-term outcome.

## Results

Between 1986 and 2012, 46 infants and children (mean age 6.6 ±2.4 months, range 1 day-17 years ) underwent either subtotal or total resection of primary cardiac tumors (13 rhabdomyomas, 10 fibromas, 9 teratomas, 8 myxomas, 2 hemangiomas, 1 rhabdomyosarcoma, 1non-Hodgin’s lymphoma and 1 lymphangioma. All had an atypical heart murmur. Left ventricular (LV) outflow tract obstruction was present in 11 children. Indications of resection were presence of hemodynamic and respiratory compromise, severe arrhythmia and risk of embolization. Approach and strategy of resection varied according to tumor location and hemodynamic status.

## Results

Mean duration of follow-up is 11.6±3.5 years. Morbidity occurred in a 5-month old girl with LV fibroma underwent LVAD implantation secondary to failure from weaning off cardiopulmonary bypass and she eventually underwent heart transplantation 17 days later. Early mortality included a 5-month old boy who underwent complete resection of rhabdomyoma located in LV. Complete tumor resection was done; unfortunately, he underwent LVAD implantation for postoperative heart failure and died on the 13th postoperative day. An 8-month old girl with 3x4cm fibroma obstructing the right ventricular outflow tract compressing the right coronary artery died of severe right heart failure on the 13th postoperative day. Late mortality (7 months postoperative) occurred in a 16-year old boy with non-Hodgkin’s lymphoma. Forty-three patients are alive and well, and are in Ross functional classification 1. All survivors were free of tumor recurrence or progression, even when the resection is incomplete.

## Conclusion

Individualized approach to tumor resection allows restoration of an adequate hemodynamic function and satisfactory long-term tumor-free outcome.

